# Investigation of the Differential Contributions of Superficial and Deep Muscles on Cervical Spinal Loads with Changing Head Postures

**DOI:** 10.1371/journal.pone.0150608

**Published:** 2016-03-03

**Authors:** Chih-Hsiu Cheng, Andy Chien, Wei-Li Hsu, Carl Pai-Chu Chen, Hsin-Yi Kathy Cheng

**Affiliations:** 1 Department of Physical Therapy and Graduate Institute of Rehabilitation Science, College of Medicine, Chang Gung University, Taoyuan, Taiwan, R.O.C; 2 Department of Physical Therapy and Graduate Institute of Rehabilitation Science, China Medical University, Taichung, Taiwan, R.O.C; 3 School and Graduate Institute of Physical Therapy, College of Medicine, National Taiwan University, Taipei, Taiwan, R.O.C; 4 Physical Therapy Center, National Taiwan University Hospital, Taipei, Taiwan, R.O.C; 5 Department of Physical Medicine & Rehabilitation, Chang Gung Memorial Hospital at Linkou and College of Medicine, Chang Gung University, Kwei-Shan, Tao-Yuan County, Taiwan, R.O.C; 6 Graduate Institute of Early Intervention, College of Medicine, Chang Gung University, Tao-Yuan, Taiwan, R.O.C; University of Utah, UNITED STATES

## Abstract

Cervical spinal loads are predominately influenced by activities of cervical muscles. However, the coordination between deep and superficial muscles and their influence on the spinal loads is not well understood. This study aims to document the changes of cervical spinal loads and the differential contributions of superficial and deep muscles with varying head postures. Electromyography (EMG) of cervical muscles from seventeen healthy adults were measured during maximal isometric exertions for lateral flexion (at 10°, 20° and terminal position) as well as flexion/extension (at 10°, 20°, 30°, and terminal position) neck postures. An EMG-assisted optimization approach was used to estimate the muscle forces and subsequent spinal loads. The results showed that compressive and anterior-posterior shear loads increased significantly with neck flexion. In particular, deep muscle forces increased significantly with increasing flexion. It was also determined that in all different static head postures, the deep muscle forces were greater than those of the superficial muscle forces, however, such pattern was reversed during peak efforts where greater superficial muscle forces were identified with increasing angle of inclination. In summary, the identification of significantly increased spinal loads associated with increased deep muscle activation during flexion postures, implies higher risks in predisposing the neck to occupationally related disorders. The results also explicitly supported that deep muscles play a greater role in maintaining stable head postures where superficial muscles are responsible for peak exertions and reinforcing the spinal stability at terminal head postures. This study provided quantitative data of normal cervical spinal loads and revealed motor control strategies in coordinating the superficial and deep muscles during physical tasks.

## Introduction

The normal spinal loads are mainly maintained and regulated by a complex multi-layered cervical muscular system during daily activities involving head movements. Superficial muscles are traditionally assumed to be the motion actuator, whereas deep muscles help to fine tune the curvature and hold an upright posture of the cervical spine [1]. Furthermore, it has been documented that the activation of cervical muscles, either for motion production or providing stability, will inevitably increase the loading of the spine [2]. With the increasing popularity and our greater reliance on smart phone and portable devices, neck problems arising from prolonged forward and downward head posture is quickly becoming a global epidemic [3]. Thus, a better understanding of spinal loads as contributed by muscle activations under environmental challenges would assist in the diagnosis and rehabilitation in preventing the development of postural induced spinal disorders.

Many kinds of techniques are proposed to estimate the in vivo muscle forces and corresponding spinal loads, which are traditionally classified into electromyography (EMG) based technique or optimization/equivalent method. A hybrid EMG-assisted optimization approach has been developed to satisfy both muscle activation measured by EMG and moment equations employed in optimization models [4], and has been used to evaluate the muscle activation in the trunk [5], cervical [6] and lumbar spine [7]. However, all muscles are predominately viewed as identical elements when functioning in neutral posture in those models, and the different characteristics of the superficial and deep muscles and their roles in regulating the spinal loads at different head postures still lacks empirical evidence.

To address these gaps in the research, the purpose of this study was to explore the changes of the cervical spinal loads and how the differential contributions of the superficial and deep muscles vary under maximal neck isometric contractions and at different head postures, utilizing the EMG-assisted optimization model. The results could facilitate better knowledge of the normal cervical spinal loads and the motor control strategies in modulating the superficial and deep muscles imposed by the performance of physical tasks.

## Methods

### Subjects

This study recruited a group of young adults between the age of 20–30 years old with a body mass index of less than 30 and without a history of neck pain or other spinal disorders that required treatment. The criteria were set in order to achieve the best homogenous healthy cervical spine as the basis for normative data. All subjects were informed of the experimental protocols before signing the participation consent forms. The experimental protocol was approved by the institutional medical research ethics committee.

### Data collection

Twenty paired muscles and one longus colli-vertical muscle were considered in this study, which included the bilateral sternocleidomastoid (comprised of sternomastoid, cleidomastoid, and cleido-occipital muscle), upper trapezius, levator scapulae, splenius (including splenius capitis-lateral, splenius capitis-medial, and splenius cervicis), semispinalis (including semispinalis capitis-lateral, semispinalis capitis-medial, and semispinalis cervicis), scalenes (including scalenus anterior, scalenus medius, and scalenus posterior), erector spinae (including longissmus capitis, longissmus cervicis, and iliocostalis cervicis), longus colli (including longus colli-vertical, longus colli-superior, and longus colli-inferior), and longus capitis. These muscles were divided into the superficial muscle group (sternocleidomastoid, upper trapezius, levator scapulae, splenius capitis, and semispinalis capitis) and the deep muscle group (scalenes, erector spinae, longus capitis and colli, and splenius/semispinalis cervicis muscles) based on the classification system utilized by Blouin and colleagues [1].”

Three pairs of electrodes of the surface EMG (Trigno Wireless, Delsys Systems, USA) were placed around the neck. The anterior electrodes were placed at lower 1/3 of the distance between the sternal notch and mastoid process to measure the activations of the sternocleidomastoid and its subvolume muscles [8]. The splenius capitis-lateral, splenius capitis-medial, and levator scapulae muscles were grouped into posterolateral muscles of the neck, whose activation levels were assumed to be the same [6], and the electrodes were located at lower 1/3 of the C7-Ear line [9]. The semispinalis capitis-lateral, semispinalis capitis-medial, and upper trapezius were grouped into posterior muscles of the neck [6], and the electrodes centered at C4 level [9].

The Trigno Wireless system does not use the reference electrode. The common mode rejection ratio was greater than 80 dB, and the band-pass filter was between 20 Hz and 450 Hz. The low-pass cutoff frequency at 450 Hz truncates the contribution from the baseline noise without removing any significant contribution from the surface EMG signal, and the high-pass cutoff frequency at 20 Hz is recommended to reduce the noise sources from motion artifacts and ECG artifact [10]. The EMG signals were recorded at 2 kHz sampling rate using 16-bit A/D board to correctly reproduce the original analog information of the sampled signal [11]. The signals were further digitally full-wave rectified, and smoothed with a 10 Hz low-pass filter to cut away the steep amplitude spikes and shape a reproducible linear envelope [12].

The head posture was measured using a 3-axis electrogoniometer (CXTLA02, Crossbow, Inc., USA) attached to the top of subjects’ heads. The electrogoniometer traces the inclination to the gravity line and offers fast-response and high-resolution measurement. The resolution of the transducer was 0.1° over the angular range of ±90°. This gravity-reference instrument was proved to show good reliability and to be feasible for clinical applications [13].

### Experimental protocols

The subjects sat on a chair with the head positioned in a neutral posture, where the line connecting the root of nose to external occipital protuberance is horizontal to the floor [14]. Their hands were placed on their thighs, and their trunk and arms were strapped firmly to a chair with a lumbar lordotic support to avoid slumped posture and to ensure a consistent baseline posture was achieved for all subjects. The experiment consists of two sets of tasks.

In the first task, the subject was verbally encouraged to perform maximal voluntary isometric contraction (MVIC) of the cervical muscles by pushing against a fixed surface for 3 seconds in the anterior, posterior, left and right directions, respectively. Before a new repetition, a 2-min rest was taken to minimize the muscle fatigue. Peak forces in each direction were measured by an S-type load cell (STC-20kgSE, Vishay, USA).

In the second task, the subjects were asked to hold the head postures in flexion, extension, and left/right lateral bending for every ten degrees and stopping at the neck terminal position. The terminal range was recognized when the subject felt the stretch of the passive tissues [15]. The order of all the tasks was randomized. The subjects were allowed to practice several times, and three repetitions were recorded for each task.

### Musculoskeletal model

The cervical spine (C0-T1) model was equipped with 24 degree of freedom and built based on anthropometric data [16]. The size of vertebrae were scaled by the vertical distance from the tragus to the actual C7 spinous process [17]. The angle of each vertebrae to the horizon in the neutral position [18], the rotation center of each vertebrae [19], and the ratio of the whole head posture to the rotational angle of each vertebrae [20] were also determined accordingly. Only the muscles that cross the center of C5-6 intervertebral disc, which is the most common levels of disc degeneration in the cervical region [21], were considered. Those muscles were divided into the superficial muscle group and the deep muscle group, and the anatomical and morphological data were obtained from a previous study [22]. The contraction force of the superficial muscles were calculated by adopting the equations based on the nonlinear EMG-muscle force relationship [7],

$$F_{i}=NAIEMG_{i}{}^{1/1.3}s_{i}\sigma_{\text{max}}+P_{i}$$ (1)

$$P_{i}=e^{\left(-10.671+7.675\cdot l/l_{0}\right)}s_{i}\sigma_{\text{max}}$$ (2)

where *F*_*i*_ was the *i*th muscle force (N), *NAIEMG*_*i*_ was the normalized average integration of EMG activity of each muscle [23], *s*_*i*_ was the muscle cross-sectional area [24], *σ*_max_ was the muscle maximal contraction force per cross-sectional area and set to be 35 N/cm^2^, *P*_*i*_ was the force due to passive elasticity from the non-linear tendon and passive elastic components of the muscle [25], *l* was the muscle length, and *l*_0_ was the muscle length at rest.

The deep muscles were assumed to be maximally activated and then adjusted by the following optimization formula [7],

$$\text{min}.\sum_{\text{i}=1}^{23}{\text{M}}_{\text{i}}^{\text{deep}}{\left(1-{\text{g}}_{\text{i}}\right)}^{2}$$ (3)

The boundary condition was that the moment exerted by muscles should balance the moment at the C5-6 level caused by the external loading as follows,

$$\sum_{\text{i}=1}^{23}{\text{g}}_{\text{i}}{\text{M}}_{\text{i}}^{\text{deep}}+\sum_{\text{j}=1}^{18}{\text{M}}_{\text{j}}^{\text{sup}}=\text{M},\;\text{and}\;{\text{g}}_{\text{i}}\geq 0$$ (4)

where the superscript ‘deep’ indicates deep muscle, ‘sup’ indicates the superficial muscles, and **M** is the external moment produced by the measured loads and the weights of the subjects' heads. The gain value, and consequently the muscle force, must be non-negative to agree the physiologically observed muscle activation patterns [7]. The decisive forces of deep muscles were calculated by multiplying the initial muscle forces with the gain values.

### Data analysis

The maximal EMG activity of each muscle was defined as the highest values of the three repetitions during MVIC among the four directions, and was used to calculate the normalized EMG activity of each muscle [26]. The spinal loads were calculated by summing the force components of all muscles and the external loads in each of the corresponding orthogonal planes [26]. The force ratio (FR) calculated as the ratio of total deep muscle forces to total superficial muscle forces were also obtained. One-way ANOVA and post hoc analysis with Bonferroni correction was used to investigate the effects of head postures on the spinal loads, sum of muscle forces, and FR in different directions. Statistical significance was considered to be at p < 0.05.

## Results

There were seventeen healthy subjects (12 males and 5 females, 24.4±1.7 years old, height: 171.4±7.5 cm, weight: 67.0±13.5 Kg) participated in this study.

### MVIC task

The measured peak exerted forces of the head from load cell were 80.7±21.1, 104.8±47.1, 62.0±24.3, and 59.5±22.2N in anterior, posterior, left, and right directions, respectively (refer to S1 Table). The normalized EMG activities of the sternocleidomastoid were around 92% during anterior MVIC. The normalized EMG activities of the splenius capitis and levator scapulae were around 90% during posterior MVIC. The normalized EMG activities of the semispinalis capitis and upper trapezius were around 50% during left/right MVIC (Table 1, also refer to S2 Table).

**Table 1:** Activation levels of the superficial muscles (% of MVIC) during the maximal voluntary isometric contraction in anterior, posterior and left/right directions.

	Sternocleidomastoid (left)	Sternocleidomastoid (right)	Splenius capitis/levator scapulae (left)	Splenius capitis/levator scapulae (right)	Semispinalis capitis/trapezius (left)	Semispinalis capitis/trapezius (right)
Anterior	91.9±5.7	92.9±5.6	59.2±23.1	62.8±18.5	27.0±17.9	39.8±26.5
Posterior	11.9±17.3	12.5±19.9	66.4±22.3	59.3±27.1	90.1±8.5	88.0±9.9
Left	40.1±22.7	20.5±24.9	69.7±15.8	21.7±13.8	49.4±18.0	36.9±19.6
Right	18.3±21.7	34.1±18.0	16.7±10.2	65.2±19.4	25.6±12.7	53.0±18.1

The greatest calculated compressive load was 1118±82 N during the anterior MVIC. The greatest anterior-posterior shear load was 153±35 N during the anterior MVIC, and greatest medial-lateral shear load was 39±17 N during the left MVIC. The sum of deep muscle forces were slightly greater during the anterior/posterior MVIC (around 415N) than those during the left/right MVIC (around 390N). The sum of superficial muscle forces was greatest during the anterior MVIC (760±77N). FR during MVIC was less than 1 in all directions with the smallest value showed in the anterior direction (0.55±0.10) (Table 2, also refer to S3 Table). The corresponding calculated neck muscle forces ranged up to 119±19 N in the sternocleidomastoid during the anterior MVIC. The greatest muscle forces during the posterior, left, and right MVIC were trapezius (61±9 N), left longus capitis (58±19 N), and right longus capitis (54±25 N) respectively (Table 3, also refer to S4 Table).

**Table 2:** Comparison of the spinal load, sum of muscle forces, and force ratio of deep muscles to superficial muscles (FR) in current study with the data estimated by the EMG-based model [26] during the maximal voluntary isometric contraction in anterior, posterior and left/right directions.

	Compression	Shear_ap	Shear_ml	MForce_d	MForce _s	FR
Present study						
Anterior	1118±82	-153±35	-1±8	414±56	760±77	0.55±0.10
Posterior	1035±238	-52±34	-3±6	417±100	704±194	0.62±0.17
Left	856±195	-137±38	-39±17	398±73	509±160	0.84±0.24
Right	809±171	-131±26	34±12	379±51	473±149	0.87±0.21
Choi et al. [26]						
Anterior	1654±308	-162±110	-28±24	-	-	-
Posterior	1372±140	-182±28	-1±30	-	-	-
Left	956±169	-74±32	-77±36	-	-	-
Right	1065±207	-98±62	89±39	-	-	-

Compression = compressive load; Shear_ap = shear load in the anterior-posterior direction; Shear_ml = Shear load in the medial-lateral direction (Positive values indicate anterior and leftward shear loads); Mforce_d = Muscles forces of the deep muscles; Mforce_s = Muscles forces of the superificial muscles; FR = Force ratio. All forces are measured in the unit of Newton.

**Table 3:** Muscle forces (unit: N) of the individual cervical muscles among participants during maximal voluntary isometric contraction (MVIC) in anterior, posterior and left/right directions. Muscles with broad areasx of attachment were represented in detail by their branches. Note that the longus colli-vertical muscle is in the midline of the vertebrae and the magnitude of the muscle force is showed in the left muscle column.

	Anterior		Posterior		Left		Right	
	Left muscle	Right muscle	Left muscle	Right muscle	Left muscle	Right muscle	Left muscle	Right muscle
Sternocleidomastoid								
i) Sternomastoid	119±19	119±19	16±12	17±3	43±9	26±20	21±15	33±3
ii) Cleidomastoid	60±10	60±10	10±6	10±7	22±9	13±8	12±8	18±6
iii) Cleido-occipital	53±9	49±12	7±6	7±7	19±9	9±8	9±7	15±6
Upper trapezius	15±11	15±12	61±9	59±0	34±0	21±10	18±8	36±11
Levator scapulae	17±12	18±14	51±18	50±20	52±21	18±11	19±10	48±15
Splenius								
i) Splenius capitis-lateral	9±6	9±7	29±11	28±5	25±11	8±5	8±5	26±0
ii) Splenius capitis-medial	8±6	8±8	31±3	28±6	25±2	9±6	10±6	29±11
iii) Splenius cervicis	30±8	30±8	26±8	27±6	15±1	15±10	10±8	11±9
Semispinalis								
i) Semispinalis capitis-lateral	10±10	12±17	55±9	51±1	30±2	18±11	14±6	30±10
ii) Semispinalis capitis-medial	4±4	6±5	50±21	50±18	24±2	16±9	15±8	30±1
iii) Semispinalis cervicis	28±8	29±7	27±1	31±25	29±17	15±10	13±9	25±22
Scalenes								
i) Scalenus anterior	64±20	63±19	8±9	8±10	23±12	13±16	11±1	20±10
ii) Scalenus medius	20±15	22±14	41±16	38±19	48±16	11±8	10±7	42±15
iii) Scalenus posterior	19±7	18±8	17±8	18±8	10±7	24±14	20±2	11±10
Erector spinae								
i) Longissmus capitis	16±4	16±4	16±14	16±13	8±5	8±5	6±4	6±4
ii) Longissmus cervicis	16±3	15±4	14±6	15±6	8±5	13±4	10±4	8±6
iii) Iliocostalis cervicis	18±2	16±4	16±7	17±7	9±6	27±14	22±14	11±12
Longus colli								
i) Longus colli-vertical	21±8	-	24±12	-	37±11	-	40±11	-
ii) Longus colli-superior	21±7	20±7	22±11	23±10	31±17	27±12	29±1	33±16
iii) Longus colli-inferior	13±3	13±3	14±5	14±4	23±8	13±3	16±6	22±8
Longus capitis	38±2	38±2	38±7	40±5	58±19	27± 3	32±1	54±25

### Static head postures

The mean terminal posture of the neck were 46.7°±8.4°, 38.7°±5.2°, 28.8°±5.4°, and 29.2°±4.6° during the flexion, extension, left, and right side bending respectively (refer to S1 Table). The normalized EMG activities of the sternocleidomastoid were significantly increased with the increasing flexion/extension angle (all p < 0.05). The normalized EMG activities of the splenius capitis and levator scapulae were only significantly increased with the increasing extension angle (all p < 0.05). The normalized EMG activities of the semispinalis capitis and upper trapezius were significantly increased with the increasing left/right side bending (all p < 0.05) (Table 4, also refer to S5 Table).

**Table 4:** Activation levels of superficial muscles (% of MVIC) during neck posture in flexion, extension, left and right lateral bending.

	Sternocleidomastoid (left)	Sternocleidomastoid (right)	Splenius capitis/levator scapulae (left)	Splenius capitis/levator scapulae (right)	Semispinalis capitis/trapezius (left)	Semispinalis capitis/trapezius (right)
Flexion						
10°	1.3±0.7	1.4±0.8	7.1±4.0	6.5±4.9	7.9±4.0	7.4±3.5
20°	1.3±0.7	1.7±1.3	6.7±3.0	6.4±4.0	8.6±4.2	8.1±3.7
30°	1.9±1.8	2.5±3.2	7.8±4.2	7.8±4.2	10.6±5.5	10.2±5.6
terminal	2.3±2.8	2.9±4.1	6.5±3.5	6.7±4.1	7.7±4.8	8.2±5.4
Extension						
10°	1.8±1.3	2.1±2.1	5.5±3.8	5.5±4.7	3.9±1.6	4.2±1.6
20°	3.8±3.3	3.8±3.1	6.5±3.9	6.2±4.0	4.3±1.9	4.5±1.7
30°	6.1±4.7	6.4±5.1	8.3±5.8	7.3±4.4	5.1±3.0	5.3±2.2
terminal	8.0±5.6	7.8±5.6	8.8±6.6	8.9±6.0	5.0±2.1	5.7±2.4
Left side bending						
10°	2.0±1.5	1.3±0.6	7.0±4.7	6.3±4.8	4.6±2.5	5.6±3.0
20°	3.4±4.6	1.3±0.6	7.0±5.1	7.0±5.3	5.7±3.1	9.0±7.0
terminal	4.8±6.8	1.5±0.8	7.9±5.6	7.2±5.0	5.8±3.1	10.3±8.8
Right side bending						
10°	1.5±0.7	2.2±1.9	7.1±4.2	6.4±6.4	5.8±3.5	4.8±1.9
20°	1.5±0.8	2.7±2.3	6.7±2.8	6.5±6.6	8.5±7.2	5.3±2.3
terminal	1.5±0.6	4.9±5.0	6.9±3.2	6.9±5.9	9.2±7.5	6.1±2.6

The compressive loads were generally increased with the increasing flexion angle. There were significant differences in the compressive loads during the neck flexion (p = 0.033) and the post hoc analysis showed that the compressive load at the terminal position was significantly greater than that at the 10-degree flexion (p = 0.038). During the extension and left/right side bending, the compressive loads were nearly independent of the posture changes. The anterior-posterior shear loads were toward posterior direction and the post hoc analysis showed that they significantly increased with the increasing flexion angle (all p < 0.001), while the trends were opposite during the neck extension (all p < 0.001). The anterior-posterior shear loads did not significantly change with the left/right side bending. The medial-lateral shear loads increased significantly toward the opposite direction during the side bending (all p < 0.001), and they were close to zero during the flexion and extension. The maximal compressive and maximal anterior-posterior shear loads under different head postures occurred at the terminal range of the flexion (634±98 N and -323±43 N, respectively), and the maximal medial-lateral shear load was at the terminal range of the left/right side bending (around 104 N). The sum of deep muscle forces and sum of superficial muscle forces were both greatest during the flexion compared to those in other neck postures. The sum of deep muscle forces were generally decreased with increasing angles during extension and left/right side bending, while that was exclusively increased with increasing flexion angle (all p < 0.01). The sum of superficial muscle forces were all increased with increasing angles in the four directions, and only that at the terminal flexion was significantly greater than that at the 10-degree flexion (p = 0.026). The mean FR ranged from 2~3 and generally decreased away from the neutral position. There were significant differences on the FR among different postures of the extension (p < 0.05) (Table 5, also refer to S6 Table).

**Table 5:** Spinal load, sum of muscle forces, and force ratio of deep muscles to superficial muscles (FR) during flexion, extension, left and right lateral bending.

	Compression	Shear_ap	Shear_ml	MForce_d	MForce _s	FR
Flexion						
10°	540±78	-176±25	0±3	401±40	163±56	2.81±1.17
20°	566±78	-221±28	0±2	425±36	177±63	2.76±1.23
30°	602±86	-274±36	0±2	445±43	209±76	2.52±1.27
terminal	634±98	-323±43	1±2	477±42	222±94	2.52±1.41
Extension						
10°	499±85	-98±16	1±1	378±54	128±43	3.26±1.06
20°	492±93	-59±13	0±2	363±64	135±43	2.94±0.96
30°	488±101	-18±11	0±2	352±76	147±45	2.58±0.84
terminal	483±113	20±11	0±1	349±85	153±54	2.53±0.88
Left side bending						
10°	516±79	-136±20	-35±5	386±47	146±48	2.96±1.12
20°	519±81	-129±24	-71±11	375±50	165±53	2.55±1.01
terminal	527±79	-127±25	-104±15	369±51	179±55	2.21±1.03
Right side bending						
10°	518±81	-136±19	36±4	385±44	148±56	2.99±1.13
20°	518±82	-130±21	72±8	378±44	160±55	2.66±0.98
terminal	520±85	-125±21	103±13	371±42	176±61	2.35±0.96

Compression = compressive load; Shear_ap = shear load in the anterior-posterior direction; Shear_ml = Shear load in the medial-lateral direction (Positive values indicate anterior and leftward shear loads); Mforce_d = Muscles forces of the deep muscles; Mforce_s = Muscles forces of the superificial muscles; FR = Force ratio. All forces are measured in the unit of Newton.

## Discussion

This study aimed to examine the changes of spinal loads and the corresponding contributions of the superficial and deep neck muscles in controlling the cervical spine during static peak efforts and changing head postures. The results showed that the compressive loads and the anterior-posterior shear loads were significantly increased with increasing neck flexion angle. The terminal range of the flexion also came with maximal compressive and maximal anterior-posterior shear loads among the different head postures. The high spinal loads could be attributed to a superficial-deep coordinated strategy during neck flexion which is distinctly different from a superficial muscle dominant strategy during other neck postures. In addition, the deep muscle forces are greater than the superficial muscle forces under various static head postures reflecting their role as the posture muscle in maintaining the optimal cervical curvature. In contrast, the superficial muscle forces are greater than the deep muscle forces for the peak exertion during isometric contractions, and the role of the superficial muscles are amplified at the terminal head posture which may contribute to reinforce the spinal stability.

The compressive loads calculated during the maximal isometric contraction in this study were lower than the previous reported data by approximately 32% in the anterior direction, 25% in the posterior direction, 10% in the left direction and 24% in the right exertion direction (Table 1) [26]. A closer examination of the muscle forces estimated in Choi and colleagues’ study were determined to be much higher (peck force of sternocleidomastoid is up to 302±89 N) than the limits of the accepted physiological muscle forces (around 137N) [22], which could subsequently lead to overestimated spinal loads and thus contrasting the results of the current study. In the current study, the maximum compressive loads were estimated to be 1118 N and 634 N during isometric contraction and different head postures, respectively. Considering the reported cervical spinal strength, i.e. 2158 N [27], the estimated compressive loads in this study are well below the ultimate strength and appears to be more consistent with the known physiological properties of the spinal structures. Furthermore, the estimated muscle forces in this study (up to 119±19 N, Table 2) were also within the reported physiological limits, with reasonable deep muscle activation and co-contraction phenomenon by considering the EMG-force relationship and by allocating the optimal weighting at the same time.

The results demonstrated that although head postures away from the neutral position are generally accompanied by gradual increasing of spinal loads, only flexion at around 46 degrees showed significantly increased compressive loads of up to 634 N. The estimated load is around three times greater than that predicted in previous study using the finite element assessment without considering the muscle forces (i.e. 49 lbs in 45 degrees of neck flexion) [3]. Greater angle of head postures in the sagittal plane are also accompanied by increased anterior-posterior shear load up to 323 N, while those in the coronal plane are accompanied by significantly increased medial-lateral shear load ranged from 35 N to 104 N. The greatest shear load in the neck accordingly could be around 1/7~1/3 body weight and it is suggested that special attention should be given to the long-term effect of not only the posture-induced compressive injuries but also shear stresses on the disc degeneration or ligament failure. To our best knowledge, this is the first study to demonstrate an estimation of in vivo cervical spinal loads with changing head postures and with isolated inspection in the coronal plane. It should be noted that the present data were determined from a series of static head postures and the loading rate during high-velocity impact conditions is not considered. Nevertheless, the directional-specific property of the spinal loads obtained from healthy adults in this study could reflect the normal stresses the neck endures during various sustained work-related postures.

It is worth noting that the terminal range of flexion came with the greatest compressive and anterior-posterior shear loads among the different head postures. This could be due to the fact that the flexion is characterized by the forward shift of the head’s center of gravity. The cervical spine is inherently prone to injury risks when in the flexed position, which is consistent with the rehabilitation focus for populations with the text-neck or forward head posture. Moreover, the loss of control from muscular fatigue or possible neuromuscular error could lead to abnormal muscle recruitments and spinal loading patterns [28], and neck disorders may eventuate. Excessive shearing forces could generated and placing stresses on the anterior/posterior ligaments, endplate displacement, anterior/posterior subluxation or dislocation [29, 30]. This was supported by the positive correlation between the incidence of neck pain and the amount of sustained neck flexion [31]. The knowledge of estimated maximal load on the spine with changing head postures may help to identify the jobs or tasks that will expose the neck to the risk of injuries, especially those requiring sustained cervical flexion at the terminal angles for prolonged periods of time.

The results of the sum of the muscle force as well as the force ratios showed that the deep and superficial muscles play different roles in controlling the cervical spine during peak exertions and different head postures. The smaller sum of the superficial muscle forces and greater force ratios of deep muscle forces to superficial muscle forces under different head postures than during peak efforts manifests the importance of the deep muscle in maintaining the spinal curvature, which coincides with the role of posture muscles [32]. On the contrary, the sum of the superficial muscle forces during peak efforts and increased sum of superficial muscle forces (i.e. decreased force ratios) away from neutral position indicated that the superficial muscles are responsible for the peak exertion and contribute to reinforce the spinal stability at the terminal range of the head postures. In addition, though there was no clear relationship between the changing force ratios and the spinal loads, the neck flexion showed increased sum of deep muscle forces with increasing flexion angle and significantly increased sum of superficial muscle forces at the terminal angle, which could contribute to the greatest compressive and anterior-posterior shear loads during flexion. The superficial-deep coordinated strategy during neck flexion is distinctly different from the superficial muscle dominant strategy during other neck postures and is worthy of further study. The current results revealed that the function of the two muscle groups should be considered as direction-specific during peak efforts and in different head postures.

Some methodologic considerations should be verified. First, the ROM in this study was the range where subjects felt mild resistance so that the EMG attachments would not be affected by the stretching of the soft tissues. It was smaller than the maximal active ROM reported in the literature (60°~70° during flexion/extension and 40°~45° during lateral bending) [33]. Secondly, the accuracy of all kinds of EMG-driven models depends on the detailed anatomy and quality of EMG measurements. In this study, forty-one neck muscles well defined in the previous studies were considered, and the superficial EMG of the selected muscles in this study is reliable and easily assessed. Thirdly, the geometrical model was based on the previously reported neck anthropometric data. Subject-specific parameters are hard to obtain and are not considered in this study. More extensive studies would be helpful to quantify the influence of aforementioned assumptions.

In conclusion, this study revealed the changing spinal loads in different head postures as well as the differential contribution of the superficial and deep muscles on the spinal loads. The significantly higher spinal loads during neck flexion posture, which could be attributed to the particularly increased deep muscle activations, may play a role in the development of idiopathic neck disorders. Furthermore, the deep muscles are evidenced to maintain the neck curvature whereas the superficial muscles are responsible for the force generation to augment spinal stability. Further studies are suggested to investigate the relation between the deteriorated muscles and neck disorders clinically, thus facilitating the design of training/rehabilitation protocols.

## Supporting Information

S1 Table Basic information. Peak exerted forces and terminal posture of the head for each subject.
(XLSX)

S2 Table EMG data during MVIC. Activation levels of the superficial muscles during the maximal voluntary isometric contraction in anterior, posterior and left/right directions for each subject.
(XLSX)

S3 Table Spinal load data during MVIC. Spinal load, sum of muscle forces, and force ratio of deep muscles to superficial muscles (FR) during the maximal voluntary isometric contraction in anterior, posterior and left/right directions for each subject.
(XLSX)

S4 Table Muscle force data during MVIC. Muscle forces of the individual cervical muscles among participants during the maximal voluntary isometric contraction in anterior, posterior and left/right directions for each subject.
(XLSX)

S5 Table EMG data at different head postures. Activation levels of superficial muscles during neck posture in flexion, extension, left and right lateral bending for each subject.
(XLSX)

S6 Table Spinal load data at different head postures. Spinal load, sum of muscle forces, and force ratio of deep muscles to superficial muscles (FR) during flexion, extension, left and right lateral bending for each subject.
(XLSX)
