# Correction: Investigation of the Differential Contributions of Superficial and Deep Muscles on Cervical Spinal Loads with Changing Head Postures

**DOI:** 10.1371/journal.pone.0153541

**Published:** 2016-04-08

**Authors:** The PLOS ONE Staff

The first author is incorrectly designated as the Corresponding Author. The correct Corresponding Author is Hsin-Yi Kathy Cheng. The correct e-mail address is: kcheng@mail.cgu.edu.tw.
